# Influences on the adoption of patient safety innovation in primary care: a qualitative exploration of staff perspectives

**DOI:** 10.1186/s12875-018-0761-2

**Published:** 2018-05-22

**Authors:** Ian Litchfield, Paramjit Gill, Tony Avery, Stephen Campbell, Katherine Perryman, Kate Marsden, Sheila Greenfield

**Affiliations:** 1 Institute of Applied Health Research, College of Medical and Dental Sciences, University of Birmingham, Birmingham, UK; 2 Warwick Medical School - Social Science and Systems in Health, University of Warwick, Coventry, UK; 3 School of Medicine, Division of Primary Care, University of Nottingham, Nottingham, UK; 4 Centre for Primary Care, Division of Population Health, Health Services Research and Primary Care, University of Manchester, Manchester, UK

**Keywords:** Patient safety, Primary care, General practice, Health services research, Quality improvement

## Abstract

**Background:**

Primary care is changing rapidly to meet the needs of an ageing and chronically ill population. New ways of working are called for yet the introduction of innovative service interventions is complicated by organisational challenges arising from its scale and diversity and the growing complexity of patients and their care. One such intervention is the multi-strand, single platform, Patient Safety Toolkit developed to help practices provide safer care in this dynamic and pressured environment where the likelihood of adverse incidents is increasing. Here we describe the attitudes of staff toward these tools and how their implementation was shaped by a number of contextual factors specific to each practice.

**Methods:**

The Patient Safety Toolkit comprised six tools; a system of rapid note review, an online staff survey, a patient safety questionnaire, prescribing safety indicators, a medicines reconciliation tool, and a safe systems checklist. We implemented these tools at practices across the Midlands, the North West, and the South Coast of England and conducted semi-structured interviews to determine staff perspectives on their effectiveness and applicability.

**Results:**

The Toolkit was used in 46 practices and a total of 39 follow-up interviews were conducted. Three key influences emerged on the implementation of the Toolkit these related to their ease of use and the novelty of the information they provide; whether their implementation required additional staff training or practice resource; and finally factors specific to the practice’s local environment such as overlapping initiatives orchestrated by their CCG.

**Conclusions:**

The concept of a balanced toolkit to address a range of safety issues proved popular. A number of barriers and facilitators emerged in particular those tools that provided relevant information with a minimum impact on practice resource were favoured. Individual practice circumstances also played a role. Practices with IT aware staff were at an advantage and those previously utilising patient safety initiatives were less likely to adopt additional tools with overlapping outputs. By acknowledging these influences we can better interpret reaction to and adoption of individual elements of the toolkit and optimise future implementation.

## Background

Primary care in the UK continues to undergo rapid changes with national initiatives leading to increasing numbers of interventions designed to meet the growing demands of an ageing and chronically ill population [1–3]. However, implementing interventions that are frequently complex and multi-dimensional [4] in an adaptive system such as primary care means change is seldom effected in a linear and prescribed manner [5–7]. In reality, successful implementation is dependent not only on elements intrinsic to the innovation but also a range of organisational and environmental factors specific to each location including existing systems, patient demographics and available resource [8–13]. This complexity has led to calls for more research to clarify the dynamics that underlie the implementation and adoption of successful innovation [14] and a better understanding of the influence of local context particularly in primary care [15].

Research suggests that innovative patient safety activities are amongst those that need to recognise the full range of influences on implementation [16] encouraging acceptability by accounting for the local circumstances of the health care setting [17, 18]. One such patient safety initiative was the NIHR School of Primary Care research funded Patient Safety Toolkit [19, 20] designed to combat the variation in safety awareness and behaviour in a modern primary care environment [21]. The multi-element toolkit was presented on a single platform to meet the challenges presented by the diversities of scale, resource and sophistication of primary care, [21–24] and intended to address shortcomings in patient safety across a range of areas such as communication, medication, and administration [19, 24–28]. Specifically the Toolkit was developed to address patient safety issues within four key areas; identifying patients at specific risk of harm, identifying gaps in safety systems, determining a practice’s safety culture, and understanding patient perspectives on safety. It comprises of six tools, a tool for rapid retrospective note review to detect patient safety incidents (the Trigger Tool), an on-line survey to assess the safety climate amongst staff (PC-SafeQuest), a questionnaire to gauge patients’ experiences of safety in primary care (PREOS-PC), a software based intervention to prevent medication related injury (Prescribing Indicators Tool), a tool to assess medicines reconciliation for recently discharged patients (Medicines Reconciliation Tool), and a checklist looking at background systems (Concise Safe Systems Checklist for General Practice) [29]. The Toolkit is currently available on the Royal College of General Practitioners’ (RCGP) website with accompanying guidance on the use and outputs of each [20].

Here we present our qualitative findings from the final phase of implementation and evaluation of the Toolkit and describe staff experiences of using individual tools alongside their views on the factors influencing the broader adoption of the Toolkit. Our findings help understand the factors that influenced this implementation and how its sustained adoption might be more precisely supported.

## Methods

### Settings/ recruitment

The Patient Safety Toolkit Project (PST) was a multiphase study conducted across five geographic areas in England [19, 20]. General Practices from the East Midlands, Greater Manchester and North Staffordshire were involved in the development of the Toolkit [19]. These were joined by practices in the West Midlands and the South Coast for the final phase when the Toolkit was implemented and evaluated across all five areas. We set out to recruit ten practices from each area with every practice implementing four of the six tools to ensure that all tools were implemented and evaluated in comparable numbers. Practices were issued an email via their local National Institute for Health Research Primary Care Research Network (latterly the regional Clinical Research Network) requesting if they would like to be involved. If they responded positively a study information pack would be issued and a meeting arranged with the local academic study lead who would discuss the project and answer any queries that emerged.

#### Distribution of the toolkit

The Toolkit consisted of six individual tools and a summary of the key attributes of each tool and the regions they were introduced can be found in Table 1. All practices distributed the PC PREOS Patient Questionnaire [30] and completed the Trigger Tool [20] (as the maximum amount of data was needed to support their further development). A combination of two of the remaining four tools, as determined by the study team, was then employed by each practice with the intention that each tool would be used at a minimum of ten practices.

**Table 1:** Description of patient safety tool kit

Name of tool	Description	Methodology	Participants	Estimated time to completion	Regions participating
Trigger Tool [20]	A system of rapid retrospective note review to allow clinicians to detect episodes of harm and patterns of error which might be occurring undetected in their practices.	Sample created of 25 random patients over the age of 75 to screen for any harm or patient safety incident. The Trigger Tool provides a framework for the case review that highlights any incidents of harm or near misses. The data is summarised to promote reflection on learning points and learning needs on an individual or practice level.	Single or multiple GPs or GP registrars	90 min	All
PC-SafeQuest [20]	An online tool which is intended to be completed by all members of the practice team allowing for a quantitative assessment of the perceived climate of safety within a practice.	Staff are invited to complete an anonymised survey on line. Once completed by a sufficient number of staff, a report can be generated summarising the findings. These are presented as a score in one of four domains, (i) workload; (ii) communication; (iii) leadership; (iv) teamwork; and (v) safety systems. These scores are then used to facilitate discussions around any issues that emerged.	All practice staff. Participation is voluntary.	10–15 min per individual.	West Midlands East Midlands Greater Manchester South Coast
Patient Reported Experiences and Outcomes of Safety in Primary Care (PREOS-PC) [20, 30]	A questionnaire to gather the experience of patients with respect to patient safety in general practice, and on patient reported safety outcomes. Questions are asked within five areas; practice activation; patient activation; experiences of patient safety events; harm; and general perceptions of patient safety.	Practice supplied with 150 envelopes containing the questionnaire, instructions for patients and a reply paid envelope. The practice will then produces the list of recipients and post the questionnaire. Completed questionnaires are returned to the authors at the University of Oxford who produce and distribute a practice specific report.	A sample of 150 patients over the age of 18 generated by the practice. A GP is expected to check that this does not include vulnerable patients.	60 min	All
Prescribing Safety Indicators [20]	Indicators involve the use of CHART (Care and Health Analysis in Real Time) software to extract data on patients at risk of medication-related injury. There are 36 in total and include prescribing related to issues such as cardiovascular and respiratory disease, immunosuppression and laboratory test monitoring.	Install CHART software, download the prescribing safety indicators from PRIMIS Hub, run the computer queries on the GP clinical system and uploading the results to CHART online. The resultant data identifies at-risk patients for the practice who then upload an anonymised version to CHART online, aggregated and shared so practices can view their results in relation to other practices.	Various (including member of study team)	60 min	West Midlands North Staffordshire South Coast
Medicines Reconciliation Tool [20]	Used to assess the quality of medications reconciliation process on discharge with a focus on vulnerable patients.	Staff populate a data collection form using the discharge document, the consultation record and the medication record of 20 patients aged 65 and over discharged from emergency hospital between 3 and 6 months ago. This data helps to assess how promptly and how accurately medication changes suggested by the hospital have been made. It also assesses the extent to which changes have been discussed with patients.	Senior staff member collecting data from records	100 min	East Midlands Greater Manchester
Concise Safe Systems Checklist for General Practice [20]	A checklist covering aspects of patient safety not covered by existing tools or legislation. Specifically relates to background systems in practices such as items relating to repeat prescriptions and logs of details of minor operations.	Completion of the checklist form by a practice manager or a senior clinician and used annually.	Senior staff member	30 min	North Staffordshire

### Data collection

**Fig. 1:**
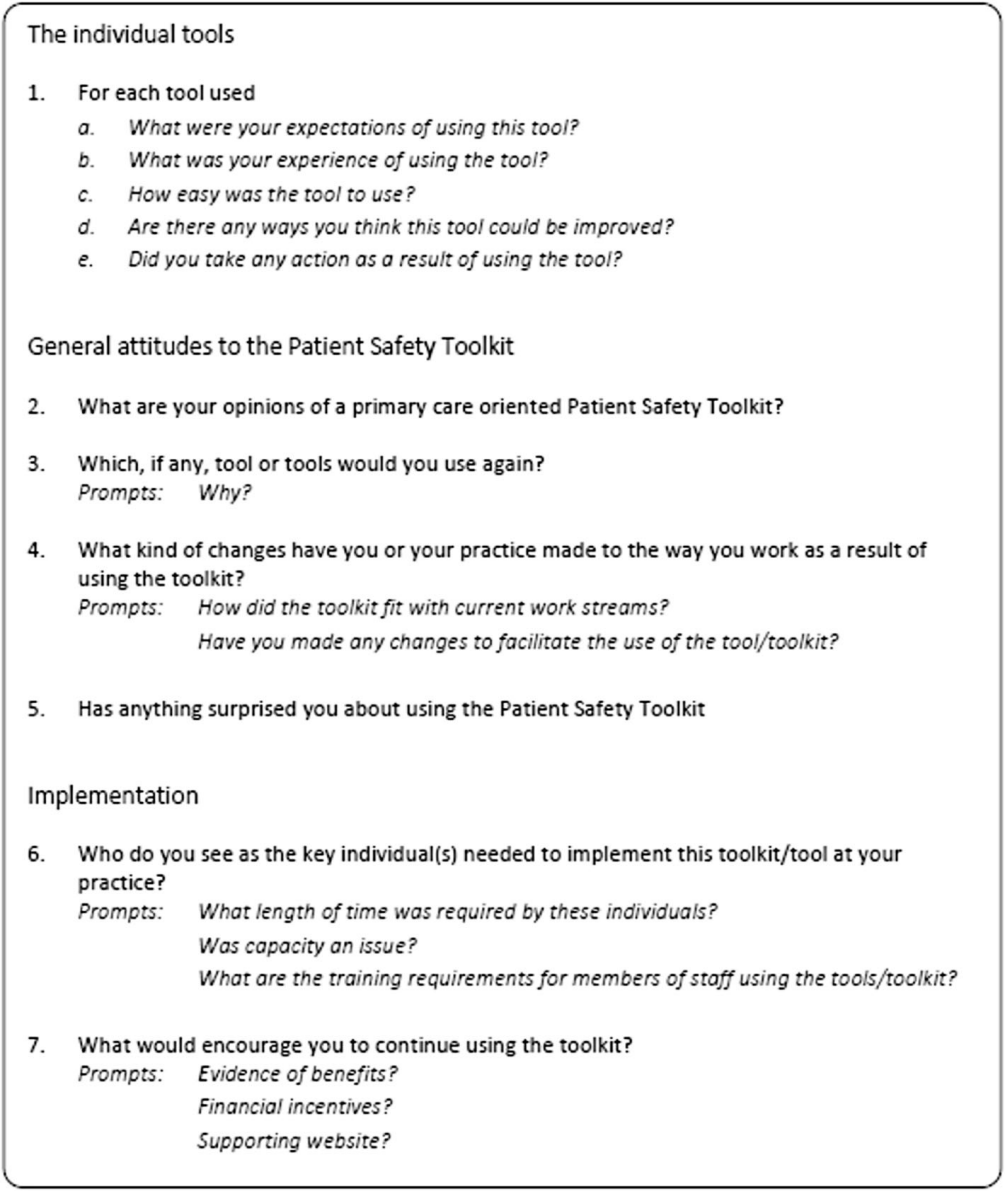
The Topic Guide

Semi-structured interviews were conducted with both clinical and non-clinical staff involved in the implementation of the PST. The interview schedule asked staff to describe staff experiences of the tools used, perspectives on the Toolkit as a whole, and its implementation in practice as summarised in Fig. 1. Due to the constraints of time and resource a combination of telephone and face to face interviews were conducted by authors IL, a research fellow employed by the University of Birmingham, KP a research fellow employed by the University of Manchester and Kate Marsden, a research associate at the University of Nottingham. All interviewers were trained and experienced in qualitative research. KP and KM had worked with the practices and interviewees in Greater Manchester and East Midlands respectively during an earlier phase of the PST project [7]. Maximum variation sampling was employed in recruiting practices to the study which meant we could explore influences on patient safety across a broad range of demographically varied cases [31, 32]. Interviews were conducted until data saturation was reached [33] and all were digitally recorded and transcribed verbatim.

### Analysis

All data were analysed using thematic analysis [34]. Three transcripts were independently coded by IL who has extensive experience of qualitative research in service delivery research and SG, a professor in medical sociology, a methodologist with expertise in qualitative research as part of mixed methods research. The emerging sub-themes were discussed and agreed upon by IL and SG and used in the subsequent analysis undertaken by IL.

## Results

Ultimately 46 practices were recruited with a range of patient characteristics and socio-economic environments [29] reflective of national averages as summarised in Table 2. Practices were predominantly from urban environments with a similar ratio of urban to rural practices found across the UK [31]. A total of 39 interviews were conducted with general practitioners (GP), a GP Registrar, practice managers (PM), practice nurses and one health care assistant (HCA). The interviews lasted between 17 and 46 min. In each case a single practice representative was interviewed except for WM07 where the interview was conducted jointly with a GP and practice manager. A breakdown of those interviewed by job role and geographical area can be found in Table 3.

**Table 2:** Average characteristics of study practices

	List Size^a^	Under 18^a^	65 + ^a^	% Non-White^b^	Deprivation Score^a^	QOF Score (2013)^a^	% Female^b^
Study practice Average/SD^c^	8824 6289	20.4% 4.7%	15.5% 7.4%	17.7% 22.6%	21.8 12.4	976.7 19.6	51.1% 5.0%
English Average	7041^a^	20.8%^a^	16.7%^a^	13%^b^	21.5^a^	961^a^	51%^b^

^a^taken from National General Practice Profiles (Public Health England) [31]

^b^taken from the GP Patient Survey July 2014 [32]

^c^The practice average and standard deviation use values that are weighted by the practice list size but the median and interquartile range use values that are not weighted by the list size

**Table 3:** Job role of those interviewed at each practice

	East Midlands (EM)	Greater Manchester (GM)	South Coast (SC)	North Staffordshire (NM)	West Midlands (WM)	Total
GP	8	4^a^	5	3	1^b^	21
PM	1	2	–	4	8	15
Practice Nurse	–	–	–	1	2	3
HCA	–	–	–	1	–	1
Total number of interviews	9	6	5	9	10	39

^a^including one GP Registrar

^b^Interviewed alongside Practice Manager

### Thematic analysis

Three key themes emerged relating to the implementation of the Toolkit: Tool Design - relating to the utility and usability of each tool; Organisational Factors relating to staff characteristics, practice culture, resource and existing safety systems; and finally Environmental Factors describing the influence of their local Clinical Commissioning Group and the broader influence of central policy. The major themes and sub-themes are summarised in Table 4.

**Table 4:** Summary of themes and sub-themes in relation to individual tools

Theme	Sub-theme 1	Sub-theme 2	Trigger tool	PC-Safe-Quest	PREOS-PC	Prescribing Safety Indicators	Medicines Reconciliation Tool	Concise Safe Systems Checklist for General Practice
1. Tool Design	1.1 Utility	Inform patient safety Training aide Provide evidence of safe practice.	Did not uncover enough learning points for those using SEA.	Provided a useful practice-wide staff perspective.	Provided a novel patient perspective.	Produced useful patient specific information.	Produced useful information.	Produced useful information that prompted reflection on safety issues.
	1.2 Usability	Format Time to completion Integration with existing systems	Was time consuming in the selection of individual records.	Completed online and easy to follow.	Resource intensive due to the addressing and packing of multiple envelopes.	Required either existing IT knowledge or additional help and support.	Straightforward to use.	Easy to use and quick to complete
2. Organisational factors	2.1 Staff training	Existing skill set	Was straightforward to use though a preference for an electronic version was expressed.	No training needed though an email address for each staff member was required.	Issues arose selecting random patients and using ‘mail merge’ to address letters and envelopes.	Staff training was required to run the software and upload the results.	No formal training required. An electronic version preferred.	No training required
	2.2 Available resource	Staffing levels, Time constraints						
	2.3 Existing patient safety approaches	Comparative effectiveness						
3. Environmental context	3.1 Clinical commissioning group	Existing initiatives	N/A	N/A	N/A	N/A	Repeated the work of a CCG initiative in one area.	N/A
	3.2 Central policy	Financial Incentives Fragmented policy						

#### Tool design

Here we describe the prima facie response to the design of the tools in relation to their utility and usability, respectively defined as the relevance and applicability of their individual and combined output, and the ease with which the tool can be used.

##### Utility

The degree to which constituent tools were able to contribute to improving patient safety was the central consideration. Staff commented on how they can be used to raise awareness of the importance of patient safety amongst staff, gain insight into safe practice from patients and non-clinical staff, quantify improvements over time, support the training of junior doctors and produce evidence of safe practice for regulators.

##### Impact on safe practice

Taken as a whole, a Toolkit requiring the input of both clinical and non-clinical staff was seen as a useful means of raising or maintaining awareness of patient safety and creating a culture where the whole work force remained attentive to its importance.

“It’s probably a part of creating this ethos of patient safety as much as anything … creating an environment where people are mindful of patient safety” *GP Registrar* – *GM03*

“…it makes you aware of what could go wrong; you know, what you need to be doing for the patient safety” *- Practice Nurse NS05*

Individual tools provided a range of perspectives on various aspects of patient safety. For example the PREOS-PC questionnaire was seen as offering a useful insight into patients’ interpretation of the concept of patient safety.

“I think probably the learning point here was “What does it mean to patients themselves?” because it may mean something else to them, and something else to us.” *- Practice Nurse WM02*

It was also felt that the PC SafeQuest tool was a practical way of supporting interaction between different staff groups, particularly between clinical and non-clinical staff. For example, one practice manager felt it an important means of managers gaining critical feedback from administrative staff that might otherwise have been missed.

“… as managers we tend to miss out on things because we are so busy with paperwork and this and that you forget your staff and it’s nice to get a feedback of the staff, of what they think and how they feel, and patient safety, communication and all that kinda stuff. So it does highlight a lot of interesting points.” – *Practice Manager WM08*

The scores produced by this tool could be compared over time to provide a quantitative measure of any changes in performance.

“So the managers obviously think we’re doing a great job, the non-managers not so convinced, looking at that [report]. Perhaps that is something we need to address. I suppose that’s one of the strengths of using this sort of approach, that you pick up things which you perhaps actually thought you were doing okay, but maybe we’re not doing as well as we could… the major utility of something like this is to repeat it and see if there is an improvement.” – *GP EM08*

##### Training aide

Staff at several practices felt the whole Toolkit would have real value as a means of training junior doctors, raising their awareness of the issues around patient safety.

“I think if I could convince a trainee to perhaps do it as a project, which I think could be really useful for them as well, then that might work really well and I think maybe I would consider doing that… in fact it’s now a requirement of completion of general practice training that they must have done either an audit or a quality improvement project and this would be ideal.” – *GP EM08*

##### Provide evidence of safe practice

In attempting to ensure delivery of care that consistently meets national standards, regulatory bodies such as the UK’s Care Quality Commission (CQC) [35] are charged with objectively measuring the performance of practices across a number of criteria. Some we spoke to felt the Toolkit would be useful in providing evidence to regulators of the work the practice do in the area of patient safety.

“So if I present this report to a [CQC] inspector, he will probably be quite surprised, ‘Where the hell did you get this from?’ and you’d actually be able to quantify it and provide some kind of qualitative interpretation as to what this actually means. They’ll probably consider it as a good or an outstanding, to be honest with you.” – *Practice Manager WM07*

##### Usability

Staff described how the design of the tools contributed to their ease of use including their composition, the length of time needed to complete them, and the degree to which they were integrated with existing systems.

##### Format

Each tool had been developed or refined during earlier phases of the study with the intention that by the final phase of evaluation the usability of novel tools such as the Concise Safe Systems Checklist had been optimised.

“The questions are well laid-out - yes/no answers and any comments that you want to make…very easy to follow.” - *Practice Manager NS04*

##### Time to completion

An important consideration was how long it took to use the tool. For example one practice manager commented how the speed with which the online survey of practice safety culture (PC-SafeQuest) could be completed was a distinct advantage.

“For me the fact that there was a good uptake suggests that it was quite straightforward to access… and the fact that it didn't take long to fill in, I mean, if it was me I, I would just look, ‘Oh God, this is taking 25 minutes. I'm not going to do it.’ So I think for a lot of people it's doable.” *- Practice Manager WM07*

By contrast the Patient Safety Questionnaire was too long for many patients to complete, certainly when they were unsure of its impact as one Practice Manager explained.

“I think it had more than 40 questions, I thought it was quite a long questionnaire so if I was a patient who had received it I may not complete it because, why would somebody complete such a long survey?” – *Practice Manager WM09*

##### Level of integration with existing systems

Staff described their preference for a tool that could integrate into existing practice software. One example was the reliance of the Trigger Tool on the manual selection of records for review. One practice manager suggested this selection process could be based on an automated algorithm to speed up the process.

“It might be better if something could be written into the clinical system like, when you go to Sainsbury’s, the random person gets a questionnaire, or the random person gets a voucher – if you get a random pop-up after, you know, X amount of patients. ‘Oh, right, I’ve gotta fill this one in, and then look back on that one.’ If it was, more like that and integrated...” – *Practice Manager WM04*

Another example was the paper-based Medicines Reconciliation Tool viewed as anachronistic by a GP Registrar in an era where patient data is increasingly held on integrated electronic systems.

“there’s an expectation amongst GPs that these sorts of tools will be...because the electronic records systems now are so good…I think it just seems a little bit strange - in a way- going back to a pen and paper system where you’re having to manually read through lists of medications and then reconcile them with manual lists on the screen.” *- GP Registrar GM03*

#### Organisational Factors

Here we describe how factors relating to individual practice organisations impacted on the attitude toward and implementation of the Toolkit. Specifically the skill set of individual staff and the resources available.

##### Staff skill set

For software based tools or those that relied on a degree of familiarity with the clinical management system the practice employed, it became apparent that a lack of information technology training limited the ability of staff to use some of the tools. One example was the Prescribing Safety Indicators tool that involved downloading and running a software package that interrogated the clinical management system for medication records then uploading the results to a secure third party for analysis. One practice manager felt the tool was too complicated and far removed from the usual roles and responsibilities of their administrative staff to use.

“I certainly wouldn’t expect the staff…they wouldn’t have a clue where to start … No, it’s got to be simple and to the point, and relative to their work, their everyday work.” - *Practice Manager WM04*

One practice nurse also described how unfamiliarity with the required software allied to the infrequent use of the tool meant they were unlikely to continue to use it.

“You can’t teach an old dog new tricks, so…it’s my knowledge of all that, you know, learning new software again and stuff. When you’re only using it once or twice, you can’t get to grips with it.” – *Practice Nurse WM02*

##### Available resource

A number of participants described how limits on staff numbers and increasing demand on staff time impacted on their ability to implement the Toolkit.

##### Staffing levels

Shortages in the number of administrative staff appeared to limit the chance of the long-term adoption of some of the tools. For example one Practice Manager felt that using her relatively expensive time to print and distribute the Patient Safety Questionnaire was not an efficient use of practice resource.

“I think if we did anything like it again, I’d ask ‘the company’ to facilitate PREOS you know? When you think what an hourly rate for a practice manager is, stuffing envelopes…because I haven’t got the manpower to pass it down.” – *Practice Manager WM04*

##### Time constraints

The pressure on the time and resource of practices of implementing a multi-faceted intervention like the Toolkit was a concern. For some, the perception that a large number of tasks required completion meant busy staff would fail to engage with the concept.

“it's a very big - it's got lots of different dimensions with GPs involved, you've got to send it out to patients, you've got internal … I think you've got to streamline it in a way… 'Oh, this sounds interesting. How much time and cost is it? I'm not interested now.” *Practice Manager WM07*

“I think you really need one sheet of paper that we can do everything on or try and streamline it down… People aren't going to do lots and lots of different tools…if you ask too much of someone they won't do it.” – *GP EM02*

Introducing any tool that took a relatively lengthy time to complete appeared problematic despite potentially valuable outputs. For example the Trigger Tool was one that involved a manual search of patient records and subsequent completion of a paper form.

“…it’s probably helpful and an important way to try and identify some of these incidents that are not...don’t lead to complaints, or they don't lead to harm. From a clinical point of view I think it’s probably just a bit too cumbersome and time-consuming to be useful and I don't think we’re going to continue using it in our practice…” – *GP Registrar GM03*

#### Existing approaches

The acceptability of individual tools was dependent on the presence and effectiveness of existing patient safety measures.

##### Comparative effectiveness

If a tools outputs were too similar to those derived from existing safety improvement approaches then they risked being judged a poor use of time. For example, one GP felt the small number of novel issues identified by the Trigger Tool in comparison to those highlighted by their Significant Event Analysis [36] were insufficient to warrant the time required for its use.

“You don't have the time to go through 10/20 sets of notes before you find one learning point because...there will be a pile of complaints, there’ll be a pile of SEAs and these are things which are prioritised because they’re more likely to lead to learning points than these sorts of trawls of triggers and things like that.” - *GP Registrar GM03*

#### Environmental context

This describes how implementation was influenced by factors relating to both the initiatives of their Clinical Commissioning Group (CCG) and also those stemming from the broader impact of national policies and guidance.

##### Clinical commissioning group

The priorities of the Clinical Commissioning Group (CCG) of each region varied. For example staff at one practice described how improvements to the discharge process orchestrated by their CCG meant the Medicines Reconciliation Tool repeated work already undertaken.

“...’cause we’ve already done things like this with the CCG, so I felt a bit like I was redoing the sort of thing that I’d already been doing.” *GP GM02*

The importance of the CCG in facilitating the sustained adoption of the Toolkit was recognised.

“…but I think it needs to go in at the commissioning side and I think we need to tie this in to a better template of use…then it actually does get built in.” *Practice Nurse EM05*

##### Central policy

Staff discussed how financial incentives influenced their decision to change ways of working, particularly when attempting to assimilate numerous policies and initiatives that emerged from a variety of sources with different agendas.

##### Financial incentives

Financial incentives have been used in primary care to encourage certain behaviours and have become relied upon as a valuable funding stream by senior staff. For some, the financial rewards of taking part in the study were a significant factor in their decision to participate.

“To be perfectly honest, it was the GP that picked up on it, from an income point of view - as another income stream. Because, you know, the way they’re pulling money off us in all directions, we’ve got to look at everything. We’re running a business, at the end of the day, so we’ve got to be doing things that are financially rewarding for the practice.” – *Practice Manager WM04*

#### Fragmented policy

Staff described how the quantity of frequently changing policies and initiatives introduced by various local and national bodies impacted on their ability to adopt further innovation.

“…[adoption is difficult] because you’re dealing with CCG, you’re dealing with NHS England, City Council, the nurses, you’re dealing with the patients, you’re dealing with your policies and procedures, you’re dealing with an audit. You got the day to day running of the surgery and then you’re going back to the action plans and the reports and all that kinda stuff. So there’s a whole sort of set of things that you need to do…” – *Practice Manager WM08*

A GP at another practice described how the number of existing work streams meant there might not be the capacity to devote the necessary time to another.

“There’s so many things, ok? That you can’t keep going ‘yet another’…all the GPs are bombarded with different practices and I don’t know, ‘ideas’ from all this and departments - I’m not sure whether they would be welcoming this. I mean I’m certain it would be useful but how much time anybody is going to spend looking in to it? I’m not certain...” *GP SC03*

## Discussion

### General findings

The concept of a Patient Safety Toolkit (PST) [20] comprised of a diverse set of tools to address a range of issues proved popular with participants. As a toolkit is was able to provide evidence of safe practice to regulators and could be used as a training aide to raise awareness of patient safety amongst Junior Doctors and the broader staff team. Of the constituent tools those that were favoured either met a distinct need and could be completed quickly and easily, such as the PC SafeQuest survey [20] or the Safety Checklist for General Practice, [20] or otherwise offered novel insights into patient safety as provided by the PREOS-PC [37].

Despite, the apparent benefit of the Toolkit, staff were hesitant about committing to its continued use due to a number of factors linked to both the practice organisation and the broader practice environment these included the need for additional staff training, its relevance in relation to existing approaches to improving patient safety, and the profusion of service initiatives from external bodies. Taken together these contextual factors impacted on the practice’s ability, capacity, and willingness to incorporate the PST into existing work streams. This relationship between innovation and the context of individual practice needs to be acknowledged and addressed if sustained adoption of this valuable patient safety resource is to be realised.

### Specific findings

#### Tool design

For any innovation to be successfully adopted it must possess clear applicability, relevance and benefit [38–42]. One tool where this was the case was the PC SafeQuest Survey [20] that provided all practice staff an anonymous platform to share their experiences of, and attitudes toward patient safety. Many previous strategies to improve quality and safety have advocated the democratisation of knowledge, skills and authority in order to successfully change systems and processes [10, 11, 43, 44] recognising the importance of an open, learning culture [45–47] and the identification of managerial “blind spots” [48, 49]. The Survey emerged as a practical and viable solution to engage all staff and increase the visibility of those issues that might have otherwise been missed by senior staff. Another tool which was well received for its’ novel perspective was the PREOS-PC patient questionnaire [37]. Though reservations were voiced about its length the resource required in its administration it was judged to provide valuable insights into patient perspectives on safety. The positive role played by patients in improving patient safety has previously been noted [50] and while the most efficient methods of harnessing patient involvement remain undefined, [51] our participants recognised the importance of understanding patient perceptions of safety and harm.

#### Organisational influences

The primary care landscape is diverse and reflected in the variation of preferences and requirements of individual practices. The skill set of staff can vary and inconsistent levels of training, particularly around IT, directly affected the capability of practices to independently implement the software based Prescribing Safety Indicators. The importance of continuous staff training to support a practice’s internal capability to deliver safer care has been described previously [38, 39, 41, 52] and smaller practices in particular can lack IT support [53, 54] limiting their use of software-based innovation [38, 55–58]. Those designing such systems should retain a socio-technical perspective that considers from the earliest design phases not only how the technical features of a system meet demand but also seeks understanding of how they interact with the working healthcare environment [4–6]. However, of larger consequence than the technical ability of staff it seems the single biggest restriction on the implementation of the Toolkit appeared a lack of time and resource. Primary care is experiencing unprecedented demand with consultation rates doubling in recent years [59, 60] in response to increasing numbers of chronically ill multi-morbid patients [61]. In the UK concerns about current and predicted shortages in the primary care workforce, are widely recognised [62] and the growing pressure on practice services not only increases the likelihood of patient safety incidents [63–67] but appears to reduce the willingness of organisations to adopt innovations or additional work streams that require any substantial amount of time or training to complete [68, 69]. For our participants this translated into an unwillingness to engage with a multi-strand toolkit or to utilise individual tools that failed to produce substantial new findings or otherwise overlapped with existing approaches to patient safety.

#### Environmental influences

Some of the existing safety initiatives being used by participating practices were introduced by their local CCG and their outputs overlapped with those of certain tools. The value of concerted CCG led initiatives may mean that their proactive (including financial) support might be the key factor in helping embed the Toolkit into existing practice systems and sustain adoption at a time when resources in primary care are so stretched [70]. The continuing financial pressures of modern primary care are leading senior staff to explore every opportunity for increasing practice income [71]. For some the financial incentives associated with piloting the Toolkit were the primary motivation for involvement, with suggestions that similar remunerations would need to be in place if they were to use the Toolkit in the future. The reluctance to unilaterally commit to its continued use might in part be attributed to the uncertainty engendered from reconciling numerous and evolving policies alongside local initiatives and directives [1, 3]. Staff described their vulnerability to a stream of frequently incoherent targets and objectives from multiple sources and this type of dynamic and complex health economy has previously been observed to reduce the willingness of senior staff to pursue innovative strategies [72].

There have been previous calls for a greater understanding of how the type of contextual influences we’ve identified here inform implementation [73–75]. Though unlikely to exist or act in isolation, [76, 77] a better understanding of the range of influences that impacted on the implantation of the Toolkit is an important step if we are to provide targeted support for this valuable patient safety resource.

## Conclusions

The experienced research team gathered data from a number of regions interviewing clinical and non-clinical staff at a range of practices until saturation was reached [33]. Not only have we identified the key design attributes of successful tools but also the inter-related contextual factors that influence the sustained implementation of complex interventions of this type. Logistical constraints meant we have so far been unable to explore the level of sustained adoption of the Toolkit by participating practices and it will be interesting to determine how the characteristics of practices and their patients influence the types of tool they continue to use.

## Abbreviations

CCG: Clinical Commissioning Group
GP: General Practitioner
HCA: Health Care Assistant
PM: Practice Manager
PST: Patient Safety Toolkit
RCGP: Royal College of General Practitioners
